# De Novo Myeloid Sarcoma as a Cause of Small Bowel Obstruction: A Case Report

**DOI:** 10.5334/jbr-btr.1353

**Published:** 2017-08-14

**Authors:** Nick Van de Voorde, Wouter Min, Rodrigo Salgado

**Affiliations:** 1 UZA, BE; 2 Heilig Hartziekenhuis, BE

**Keywords:** Isolated Myeloid Sarcoma, Granulocytic Sarcoma, Chloroma, Small Bowel Obstruction, Acute Myeloid Leukemia

## Abstract

Myeloid sarcoma (MS) is an extremely rare disease, closely correlated to Acute Myeloid Leukamia (AML) and presenting as a tumoral lesion in potentially any anatomic location. It is seen either concomitant with AML, during remission, or more seldom, prior to any detectable haematological abnormality. While MS remains a difficult diagnosis, this rare tumor must be included in the differential diagnosis of atypical, local obstructive abdominal processes, especially when coinciding with a myeloproliferative disorder. We present a case report of an otherwise healthy young patient with small bowel obstruction due to an invasive ileal mass, histologically diagnosed as a myeloid sarcoma.

## Introduction

Myeloid Sarcoma (MS) is an extremely rare tumoral lesion, with an infaust prognosis. It presents as an extra-medullary manifestation of a myeloproliferative disorder, strongly correlated with acute myeloid leukemia (AML). MS can occur at all ages, in any possible part of the body. Presentation and symptoms are therefore linked to its anatomical site. In our case, a patient, without previous haematological medical history, presented with an obstructive small bowel lesion, after resection histologically diagnosed as a myeloid sarcoma.

## Case Report

A 46-year-old man with no relevant medical history presented at the emergency department with nausea and a vague epigastric abdominal pain. An initial ultrasound examination demonstrated an ileus of the small intestine with small bowel wall distention mainly in the peri-umbilical region.

**Figure 1:**
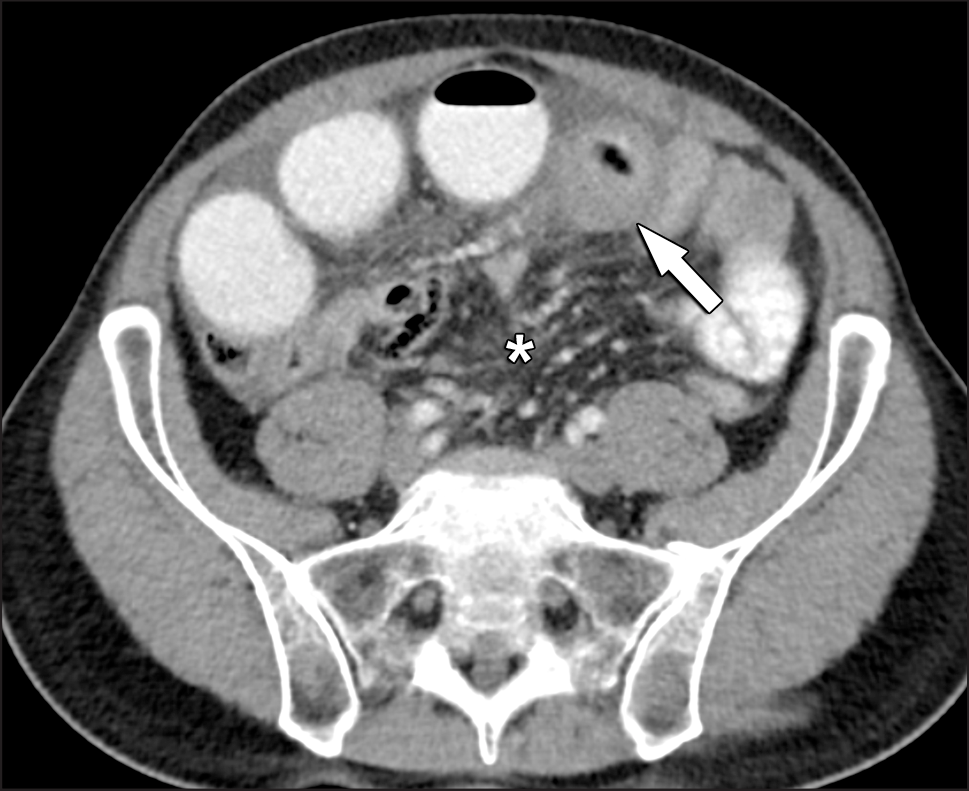
Axial view of contrast-enhanced CT reveals circumferential small bowel wall distention (arrow) and engorged mesenteric vessels (*).

**Figure 2:**
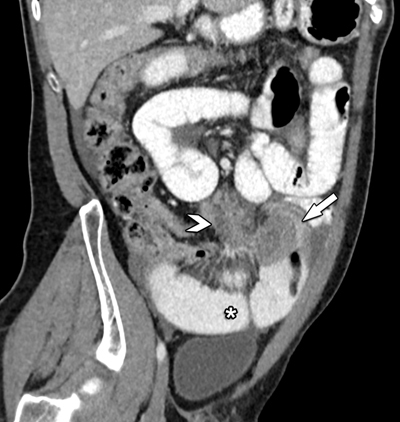
Oblique coronal view of contrast-enhanced CT shows the close relation between the mesenteric mass (arrowhead) and its extension towards the small bowel (arrow). Note the distended small bowel loops (*).

Computed tomography (CT) confirmed a large mesenteric tumoral mass extending towards the ileum, where circumferential small bowel wall invasion caused intestinal obstruction (Figures 1 and 2). There was only a moderate amount of ascites. No signs of peritoneal carcinomatosis, distant metastases or free intra-peritoneal air were present.

**Figure 3:**
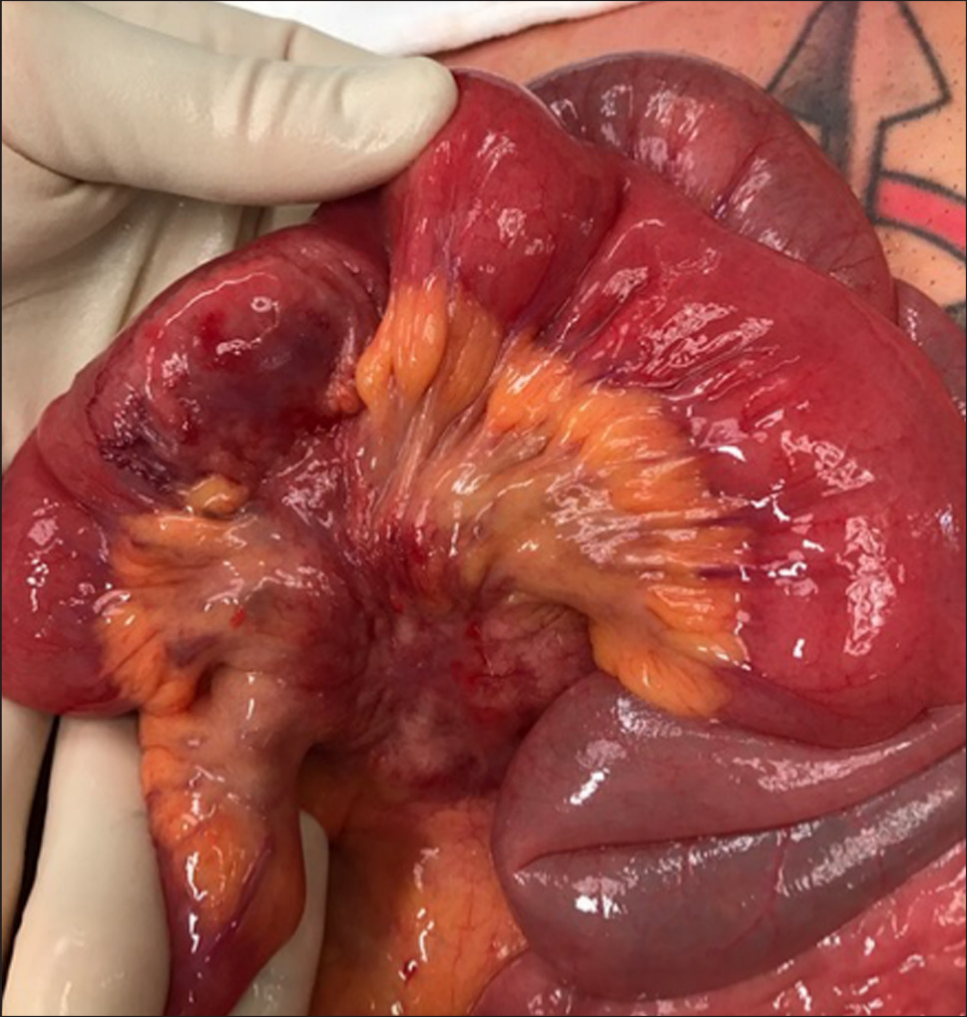
Peroperative image reveals the small bowel wall invasion and mesenteric mass.

The patient was subsequently referred for surgery, revealing an obstructive tumoral lesion in the ileum and a mass in the adjacent mesentery (Figure 3). There was no peritoneal spread of disease. The affected ileum and mesentery were resected and an entero-enteric anastomosis was made.

**Figure 4:**
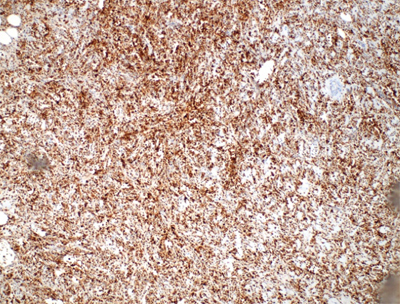
Immunohistological staining reveals highly positive lysozyme markers.

The pathology examination confirmed an ileum tumor five centimeters in length, invading all layers of the bowel wall and a second, mesenteric mass six centimeters in length. Two out of nine lymph nodes were positive. On histology, the resected mass consisted of atypical cells with a high mitotic activity and an increased nuclear-cytoplasmatic ratio. Immunohistologic staining showed a high Ki-67 expression and highly positive myeloid markers such as MPO, CD-43, CD-117 and Lysozyme (Figure 4). As such, the diagnosis of myeloid sarcoma was made.

The patient was referred to a tertiary center for further haematological work-up. Bone marrow aspiration showed no tumoral invasion. Induction chemotherapy was initiated and a stem cell transplantation was scheduled. PET-CT evaluation and haematological follow-up confirmed disease remission at the date of this publication.

## Discussion

Intrinsic tumors of the small bowel only account for approximately 1–6% of all gastro-intestinal neoplasia’s. As much as 50% of all small bowel tumors are metastases. The most common primary malignant lesions are carcinoids (44.3%) followed by adenocarcinoma’s (32.6%), lymphoma’s (14.7%), GIST’s (7.2%) and sarcoma’s (1.2%) [1]. Benign small bowel lesions include GIST, hemangioma, leiomyoma and lipoma.

In contrast, a myeloid sarcoma, also referred to as granulocytic sarcoma or chloroma, is not a primary small bowel malignancy, but rather an extra-medullary manifestation of a myeloproliferative disorder. It is mainly associated with AML and to a lesser extent with chronic myeloid leukaemia, myeloid proliferative neoplasm and myelodysplatic disorder.

MS occurs at all ages, yet 60% are found in pediatric patients [2], in contrast to AML which has an increasing incidence with age. MS encompasses proliferation of immature myeloid blasts which can occur at any anatomical site of the body, with (by definition) exception of the bone marrow. As such, symptoms vary according to the anatomic site of involvement [3]. However, up to 50% of patients have no symptoms [2]. The most frequently reported affected tissues include skin, bone, lymph nodes and soft tissue [4]. While MS can precede the diagnosis of AML by several months (isolated or de novo MS), it is mostly encountered concomitant with or during the course of treatment for AML. It affects 2.5–9% of adults with AML and is expected to increase in incidence due to prolonged survival with improving chemotherapy regimens and allogeneic hematopoietic stem cell transplantation [3,4,5]. Isolated MS on the other hand, has an incidence of two in one million adults [2]. Considering the extreme rarity of this disease and as it shares similar positive immunohistological patterns with other haematological neoplasms, a clear diagnosis is often particularly difficult. Frequent histological misdiagnoses therefore include non-Hodgkin lymphoma, histiocytic lymphoma, thymoma, myeloma, eosinophilic sarcoma, extra-medullary haematopoiesis and MALT [6].

Imaging features on CT and MR are fairly non-specific, including variable morphology and enhancement, possibly mimicking abscess formation, meningioma or lymphoma [2,7]. Multiple synchronous localisations, recurrence and osteolytic bone invasion have been described. On an abdominal level MS typically reveals plaque-like or nodular bowel wall thickening and/or mesenteric soft tissue mass and local invasion of affected tissue architecture [2]. MS can cause varying degrees of obstruction reflecting in distended proximal small bowel loops. Imaging features in other anatomical locations are also non-specific, but a solid, enhancing, FDG-PET/CT-positive mass with multiple or unusual localisations in combination with a myeloproliferative disorder, is highly suspicious for MS [7]. MRI is the imaging modality of choice when the central nervous system is involved. PET-CT can be of added value for evaluating synchronous localisations and monitoring disease remission [8].

MS is associated with a poor prognosis. Without chemotherapy all cases of isolated MS progress to AML within one year [9]. A retrospective study pooling all 345 reported MS patients in the United States between 1973 and 2010, showed a median overall survival of eight months for isolated MS as compared to five months for non-MS AML, which can be explained by a diagnosis in an earlier disease stadium, not only owing to lead time bias, because the survival advantage maintains up to five years [9]. A more recent multicentre study analysing 48 MS cases, measured a median OS of 16.7 months and a five-year survival rate of 33% [10]. Allogeneic stem cell transplantation significantly increases prognosis [10].

In conclusion, a myeloid sarcoma is a rare extra-medullary manifestation of a myeloproliferative disorder and can cause abdominal obstruction when presenting as a mesenteric and/or small bowel mass. While it can precede an AML, it must be considered in the differential diagnosis of all patients presenting with a non-specific mass and a history of haematological malignancy.
